# Low-cost (<€5), open-source, potential alternative to commercial spectrophotometers

**DOI:** 10.1371/journal.pbio.3000321

**Published:** 2019-06-12

**Authors:** Vasco Ribeiro Pereira, Bill Stephen Hosker

**Affiliations:** 1 Research and Development, RVP Didática, Sintra, Lisbon, Portugal; 2 Department of Biology, Bath Spa University, Bath, United Kingdom

## Abstract

Spectrophotometry is a fundamental technique in many areas of science, with many applications and uses. The cost of spectrophotometers has acted as a barrier on the teaching and use of the technique. Here, we provide open-source plans to a 3D-printed cuvette holder with an interchangeable narrow–spectral bandwidth light-emitting diode (LED) block that can be used in conjunction with a smartphone’s ambient light sensor (ALS) to perform spectrophotometry. A Lego version with an interchangeable LED block is also presented. Results from the smartphone spectrophotometer in comparison with commercially available spectrophotometers demonstrated functionality, and the model may have many applications, especially in indirect spectrophotometry, such as in the protein assay shown here. The plans for the 3D-printed model are freely available on GitHub, as are editable files to allow customisation by users. We would encourage users to share adaptations with the scientific community.

## Abstract 2

The cost of spectrophotometers has acted as a barrier to the teaching and use of spectrophotometry; this Community Page article provides 3D printed plans for a cuvette holder with an interchangeable LED block that can be used with a smartphone’s Ambient Light Sensor (ALS) to perform spectrophotometry for less than €5.

## Introduction

Spectrophotometry is a versatile and widely used method. One of the most fundamental uses of spectrophotometry is the use of Beer’s law to determine the concentration of an absorbing species from the measured absorbance. This quantitative use has applications in many subject areas including healthcare, pharmaceuticals, agriculture, environmental monitoring, food production and analysis, and research. One barrier to the further use of the technique is cost, with the cost of a spectrophotometer typically over €1,000. This is prohibitive to the study of the technique at most schools and in the application of the technique in many parts of the world. The typical lack of portability and requirement for mains electricity for most spectrophotometers is also a barrier to use in different areas outside the laboratory. The described model is an attempt to provide a simple, cheap, functional, open-source, basic spectrophotometer that is robust and portable, requires no expertise in electronics to construct, and can be adapted by users in ways to suit different applications.

There have been a number of attempts to create low-cost do-it-yourself (DIY) spectrophotometers, including with the use of narrow bandwidth–emitting light-emitting diodes (LEDs) as well as those based on diffraction of a polychromatic light source. Many previously published models were primarily aimed at acting as a learning tool for the principles of spectrophotometry [1–5] rather than alternatives to commercially produced spectrophotometers. The use of LEDs has been tested using other methods of detection with good adherence to Beer’s law shown, although those that have published results that were comparable to commercial spectrophotometers have been relatively complex projects requiring familiarity with electronics [5–9].

The use of a smartphone’s ambient light sensor (ALS) to detect and measure light in conjunction with smartphone software to calculate absorbance values has obvious advantages: it is a technology that for many is preexisting, it requires no need for electronic knowledge, and it has a useful range of wavelengths detectable (on models tested here, the detectable range covers at least 400–933 nm). The portability and robustness of smartphones, alongside long battery life, are also benefits to use of a smartphone in DIY scientific equipment, especially outside of a laboratory. Smartphones have been utilised previously in the construction of a low-cost microscope [10] and also in the potential acoustic detecting, identifying, and mapping of mosquito species [11], exploiting the portability and robustness of smartphones.

A potential limitation of the use of LEDs in spectrophotometry is in the relatively broad spectral bandwidth of light emitted. It is often stated that obedience to Beer's law only strictly applies when measurements are made with monochromatic source radiation. In practise, the light emitted from many spectrophotometers is not truly monochromatic but a narrow range of wavelengths [12, 13]; thus, the traditional, functional distinction between colourimetry and spectrophotometry is blurred. The narrower the spectral bandwidth of a light source, the greater the ability of the spectrophotometer to distinguish between molecules present with similar absorbance spectra, and so the resolution also increases, allowing for an accurate determination of the molar absorptivity constant [12, 14]. Narrow spectral bandwidth is obviously vital for some applications but not all. Indirect methods, in which a signal molecule is formed that has a high molar absorptivity and/or is generated in high concentration, are less likely to be affected by relatively wide spectral bandwidths than direct spectrophotometric analysis of complex solutions containing molecules with similar absorbance spectra at similar concentrations/molar absorptivities.

The model described here is offered as a contribution to the increasing amount of free open-source hardware provided by the community of science makers/DIYers [15, 16]. The plans and editable files are available on GitHub (https://github.com/VascoRibeiroPereira/phone-spectrophotometer), and we would encourage users to suggest ideas for this project by creating ‘an issue’ (instructions: https://help.github.com/en/articles/creating-an-issue) and/or adapting and sharing modified designs within the scientific community and beyond, using this or other environments. To keep the cost of the described smartphone spectrophotometer as inexpensive as possible and as open to all, commercially available LEDs with specific wavelengths stated in the descriptions (as peak emission wavelengths) were sourced from retailers on Amazon. The cost of the LEDs used in the tests shown here was <€0.10 each, although they could not be bought singularly (see S1 Table for bill of materials).

## Description of smartphone spectrophotometer

**Fig 1:**
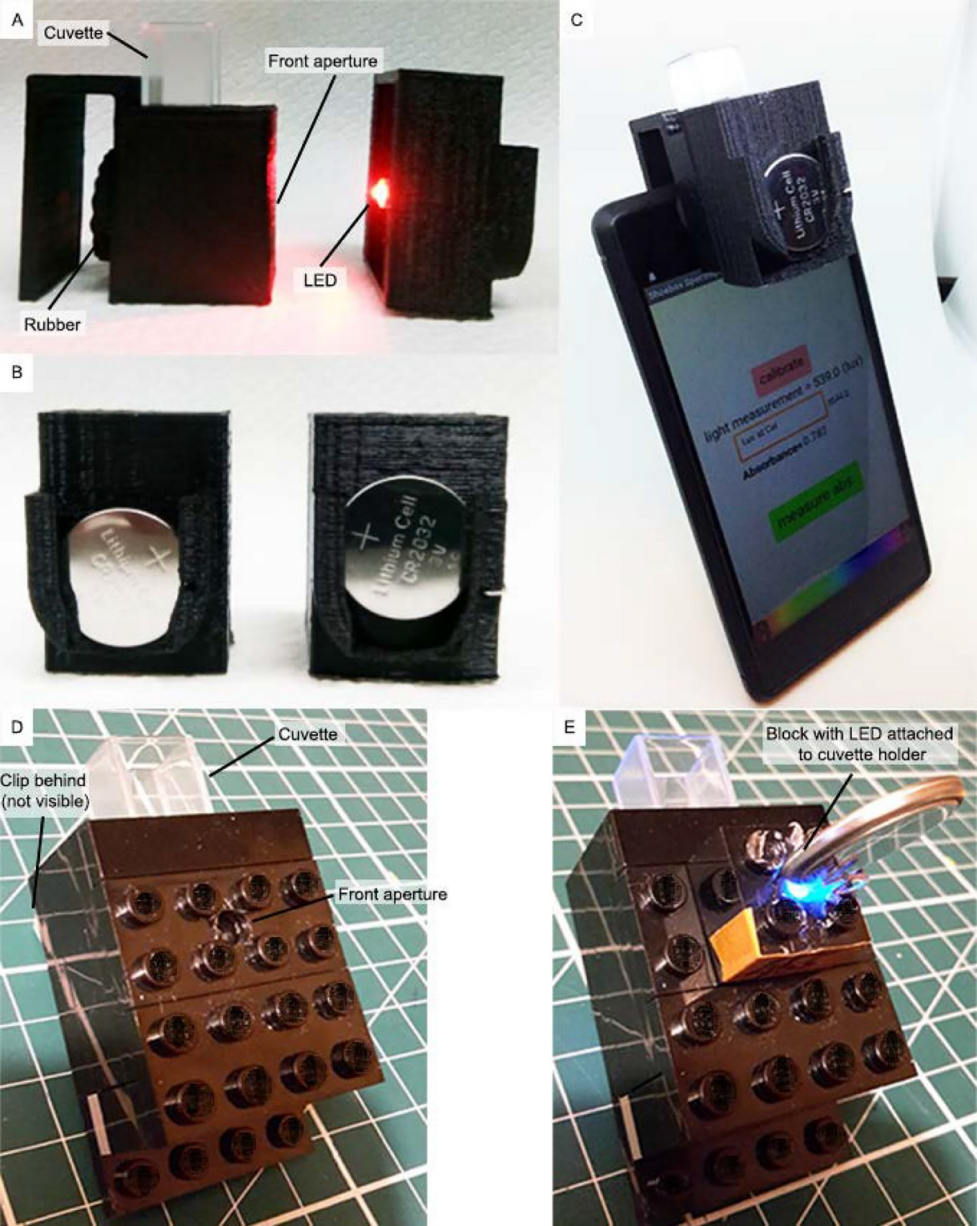
A 3D-printed version (A–C) and Lego version (D and E) of the cuvette holder. (A) Cuvette holder and interchangeable part with narrow-bandwidth LED. (B) shows the battery mechanism of the interchangeable part (pushed up for off, pushed down for on). (C) shows the holder in place. (D) shows Lego cuvette holder with front aperture, which aligns with the interchangeable Lego block with LED inserted (E). LED, light-emitting diode.

Fig 1 shows the cuvette holder and the interchangeable part with an LED and CR2032 battery. Both parts are 3D printed in polylactic acid (PLA) (diagrams shown in S1B Text). The cuvette holder has a rubber piece with a hole (Fig 1A), allowing the equipment to be well positioned and fixed and preventing outside light to reach the sensor. As in the Lego version (Fig 1D and 1E), the front aperture lines up with the rear aperture and, when attached, with the smartphone’s ALS (Fig 1C). Fig 1B shows the switch mechanism used in the interchangeable part—when the battery is up, the LED is off, and when the battery is down, it touches the mounted LED legs, turning it on. The app used to calculate absorbance from the light measurements (‘Shoebox Spectrophotometry’) is available for free (and advert free) from Google Play store [17]. The real-time light measurement on the app allows the correct positioning of the cuvette holder, with the LED fully aligned with the ALS. When the LED was switched off, the real-time light measurement should be 0 to demonstrate that the sensor is sufficiently protected from external light when attached to the phone. Full assembly instructions are available on GitHub (https://github.com/VascoRibeiroPereira/phone-spectrophotometer).

### Similarity of results to commercial spectrophotometers

To assess the functionality of the smartphone spectrophotometer, 3 calibration graphs were constructed for 3 molecules, which absorb maximally at wavelengths suiting the emission spectra of the 3 LEDs: crystal violet (587 nm), methyl orange (465 nm), and p-nitroaniline (400 nm) (emission spectra of the LEDs are shown in S1A Text, and the absorbance spectra of the solutions are shown in S1C Text).

**Fig 2:**
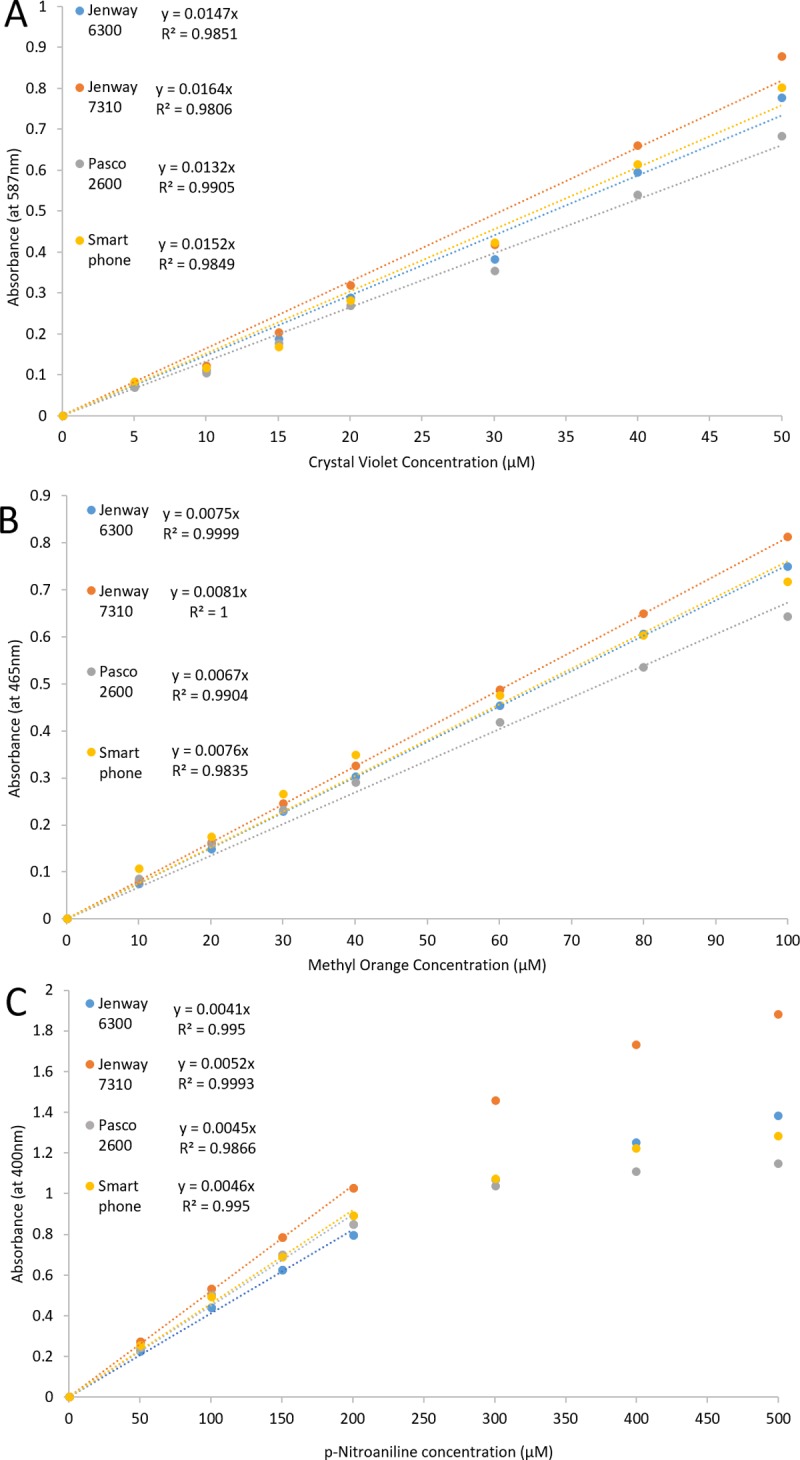
Calibration graphs for crystal violet (A), methyl orange (B), and p-nitroaniline (C) from 3 commercial spectrophotometers and the smartphone spectrophotometer. Points are from a single observation of the same solution for all spectrophotometers. Concentrations of p-nitroaniline demonstrating negative deviation (at concentrations > 200 μM) have been eliminated from the trend lines equations for all spectrophotometers. Data are available in S1 Data.

Fig 2 shows similar absorbance, trend line equations, and R^2^ values for the low-cost smartphone spectrophotometer compared with 3 other commercially available spectrophotometers in calibration graphs for crystal violet (Fig 2A), methyl orange (Fig 2B), and p-nitroaniline (Fig 2C) at 587 nm, 465 nm, and 400 nm, respectively. There is negative deviation from Beer’s law seen for all spectrophotometers at concentrations of p-nitroaniline above 200 μM, and the smartphone spectrophotometer showed similar deviation to the commercial spectrophotometers (Fig 2C).

## Functionality assessment with a protein assay

**Fig 3:**
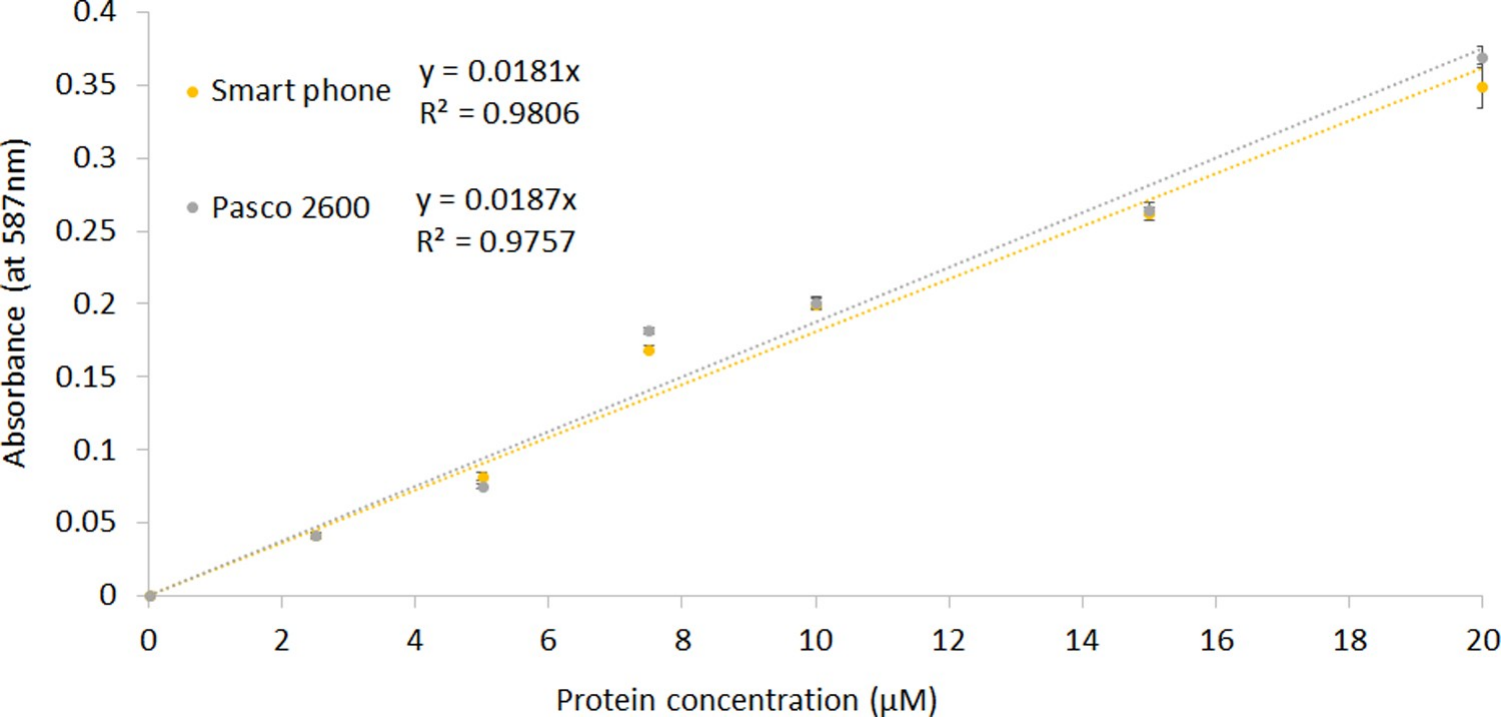
Calibration graph for protein using the Bradford assay for smartphone spectrophotometer and Pasco 2600 spectrophotometer. Points are means of 4 observations; error bars represent ±1 SD. Data are available in S1 Data.

To further assess the utility of the smartphone spectrophotometer, a protein assay was used. The Bradford assay [18] is a widely used method for the determination of protein concentration. It exploits a change in absorbance spectrum when Coomassie Blue (in Bradford reagent) reacts with proteins in solution, causing the maximum absorbance to move from approximately 465 nm to approximately 590 nm. As shown in Fig 3, individual absorbance values, trend line equations, and R^2^ values were similar for the smartphone spectrophotometer and the Pasco 2600 at 587 nm.

## Overview

Here, using calibration data from 3 different molecules tested, we have shown that results at 3 different wavelengths from the smartphone spectrophotometer are similar to the results from 3 commercially available spectrophotometers. Results from the protein assay were also in agreement with a commercially available spectrophotometer.

Together, these results suggest that the model shown here shows functionality. The low cost, relatively easy construction, and portability would make this model useful for many applications. It would also be of potential use for a range of environments/situations, such as field work in which a typical, mains-powered, relatively large commercial spectrophotometer would not be of use. The relatively low cost of the components and availability of a range of LEDs that emit maximally at different wavelengths could allow many more people to be introduced to and use spectrophotometric analysis. This could include in resource-challenged countries, where other open-source, easily constructed equipment has helped provide pipettes and micromanipulators [16]. Although many potential users may not have access to equipment to allow determination of emission spectra, this information is often provided by manufacturers, and this would allow users to determine the potential use of an LED for their intended application.

### Limitations and potential modifications

The relatively broad spectral bandwidth (shown in S1A Text) and decrease in light intensity due to battery drainage (S1D Text) would limit some of the uses of the smartphone spectrophotometer, especially those relating to direct spectrophotometry of complex solutions (such as biological fluids) or measurement of changes in absorbance over time (such as enzyme activity assays). This battery drainage limitation may be ameliorated by ensuring absorbance measurements are taken close in time to the calibration, as they were in the presented work, and/or by inclusion of a resistor (S1D Text). There is an initial decrease in intensity even with the inclusion of a resistor, so if using this model for applications in which measurements over time were important, it would be advisable to switch the LED prior to use to allow the intensity to stabilise. Even with these limitations, results here suggest the smartphone spectrophotometer can be used for indirect spectrophotometry, in which a reaction is utilised to generate a signal molecule at high concentration, or with direct spectrophotometry with molecules with high molar absorptivity at a known wavelength to gain results comparable to commercially available spectrophotometers.

## Conclusions

Results suggest the smartphone spectrophotometer here could be a useful tool for many spectrophotometric applications, despite the relatively broad spectral bandwidth of light emitted from the LEDs tested.

Because of the effect of battery drainage on light intensity, absorbance measurements should be taken in close temporal proximity to calibration, and/or an appropriate resistor should be used.

## Materials and methods

### 3D modelling and printing

The 3D models were made in SketchUp Make [19] and exported as surface tessellation lattice (stl) files for the phone/cuvette holder and LED block.

The stl files were imported to Ultimaker Cura software [20], and the G-code files were made using the described specifications: 0.2 mm of layer height, 2 mm of wall thickness, 0.8 mm top/bottom thickness, 20% infill density, 210°C printing temperature, retraction enabled, 50 mm/s print speed, and without supports. The total printing time for both models was 80 minutes.

The 3D printer used was a RepRap Micro Delta Original [21], which uses a fused deposition modelling process and a Traffic Black PLA filament (RAL 9017) from Fillamentum Manufacturing Czech s.r.o. [22].

Under the specified printing conditions, 20 g of material was used, and very little postprocessing was needed after the printing process, mostly due to a little excess of the fused plastic material. Plans and more detail are provided in S1 Text.

### Spectrophotometric analysis

LED emission spectra were measured using a Pasco PS-2600 wireless spectrometer and fibre optic cable (PS-2601), with the detector 1 cm away from the light source. Absorbance spectra of the solutions were measured using a Pasco PS-2600 wireless spectrometer, with results exported into Excel for analysis.

Cuvettes were polystyrene from Fisher Scientific (product code 11537692).

Bradford reagent (B6916) and bovine serum albumin (BSA) (A7906) were from Sigma-Aldrich.

Batteries and LEDs used are indicated with prices and sources in the bill of materials in S1 Table.

### Calibration graph construction

Dilutions of different concentrations (in deionised water) were made and placed in cuvettes. The same samples (in the same cuvettes) were used in the 3 commercially available spectrophotometers (Jenway 6300, Jenway 7310, and Pasco 2600) as well as the smartphone spectrophotometer. Deionised water was used as a blank to calibrate all spectrophotometers (including the smartphone spectrophotometer).

### Bradford assay

Solutions of different concentrations of BSA were created in deionised water. In total, 1.5 mL of the solutions was mixed with 1.5 mL of Bradford reagent and allowed to incubate for 10 minutes before the absorbance was measured at 587 nm on the smartphone spectrophotometer as well as a commercially available spectrophotometer (Pasco 2600). A solution of 1.5 mL deionised water and 1.5 mL Bradford reagent was used as a blank.

## Supporting information

S1 Table BOM, bill of materials. (DOCX)

S1 Text Emission spectra of LEDs used, absorbance spectra of solutions, further detail on construction of 3D-printed model, and discussion of adaptations including data for light intensity over time with and without a resistor. LED, light-emitting diode.
(DOCX)

S1 Data Data for figures shown. (XLSX)

## Abbreviations

ALS: ambient light sensor
BSA: bovine serum albumin
DIY: do-it-yourself
LED: light-emitting diode
PLA: polylactic acid
stl: surface tessellation lattice
