# Supplementary material for: Low-cost (<€5), open-source, potential alternative to commercial spectrophotometers
Source: PLoS Biol. 2019 Jun 12;17(6):e3000321. doi: 10.1371/journal.pbio.3000321 (PMC6590830; doi:10.1371/journal.pbio.3000321)
Supplement: S1 Table — (DOCX) [file pbio.3000321.s001.docx]

**S1 Table. Bill of materials**

| Material | Source | Price |
| --- | --- | --- |
| 400nm LED | <https://www.amazon.co.uk/dp/B008U1OEIK/ref=pe_3187911_185740111_TE_item> | €0.59 for 10  €0.06 each |
| 465nm LED | <https://www.amazon.co.uk/dp/B00SO1RQI6/ref=pe_3187911_189395841_TE_3p_dp_1> | €1.04 for 50  €0.03p each |
| 587nm LED | Part of set from Maplins, Bath (now out of business. Also available from <https://www.reichelt.com/gb/en/set-led-30-pieces-s36-p119303.html?&trstct=pol_0> | €1.76 for 30  €0.06 each |
| App | Free (and advert free) available from Google Play <https://play.google.com/store/apps/details?id=appinventor.ai_billhosker.Shoeboxspec> | NA |
| Smartphone | Pre-existing for many | NA |
| CR2032 battery | Poundland, Bath | €1.17 for 8  €0.15 each |
| PLA Model (Cuvette Holder and LED Block) | Self-made by 3D printing | €0.56 |
