# Supplementary material for: Low-cost (<€5), open-source, potential alternative to commercial spectrophotometers
Source: PLoS Biol. 2019 Jun 12;17(6):e3000321. doi: 10.1371/journal.pbio.3000321 (PMC6590830; doi:10.1371/journal.pbio.3000321)
Supplement: S1 Text — LED, light-emitting diode. (DOCX) [file pbio.3000321.s002.docx]

**Supporting Information**

As shown in S1A Text, the Full Width Half Maximum (FWHM) spectral bandwidth, measured using Pasco 2600 spectrophotometer was 14nm, for 400nm LED, 18nm, for the 465nm LED and 19nm for the 587nm LED. This is similar for the measurements for similar LEDs shown previously [1]. This compares to a stated spectral bandwidth of 8nm for the Jenway 6300 spectrophotometer [2] and 6nm for the Jenway 7310 [3], in the visible range.


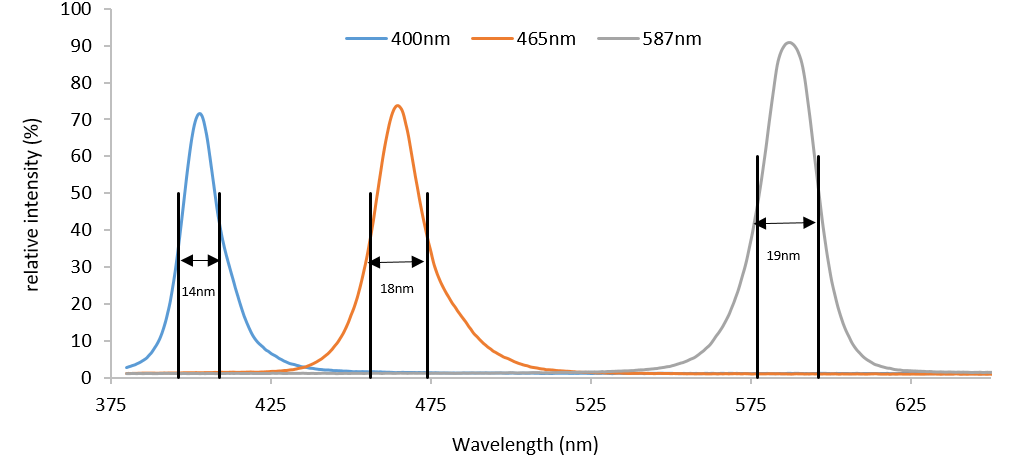


S1A Text. The emission spectra of the LEDs used in the tests, with Full Width Half Maximum (FWHM), spectral bandwidth, shown. Data available in S1 Data.

The relatively large spectral bandwidth of the LEDs did not seem to have greatly affected the results for the molecules tested, as evidenced by the similarity of individual absorbance values and linear regression equations, compared to the 3 commercially available spectrophotometers tested. While monochromatic light (or at least the narrowest spectral bandwidth possible) is obviously the ideal for spectrophotometric analysis (and necessary for some applications), the results here, together with the acknowledgement of lack of true monochromicity in many commercially available spectrophotometers indicate this ideal is not required for many applications. If an application requires a narrower bandwidth, there is the possibility of using filters to reduce the bandwidth of light emitted, although as indicated by results presented here, this would not be necessary for many applications.

**3D printing method**

The presented model may be obtained from <https://github.com/VascoRibeiroPereira/phone-spectrophotometer>. The model is composed of 2 blocks: the cuvette holder and the LED/battery holder, both of them modelled in SketchUp Make.

The cuvette block was modelled with a front aperture that tightly accommodates a 5mm LED and a rear aperture of 3mm diameter, that must be aligned with the phone ambient light sensor. The dimensions of the models where optimized to accommodate a 1cm cuvette, LED and a CR2032 battery with minimum interference of light from the outside. For this propose the minimum wall thickness used was 3mm in black Polylactic Acid (PLA).

After the print, a holed rubber was glued to the section of the rear hole allowing a better aligning and stability of the model with the phone ambient light sensor and diminishing the external light. The chosen LED is also mounted as evidenced in S1B Text (B)


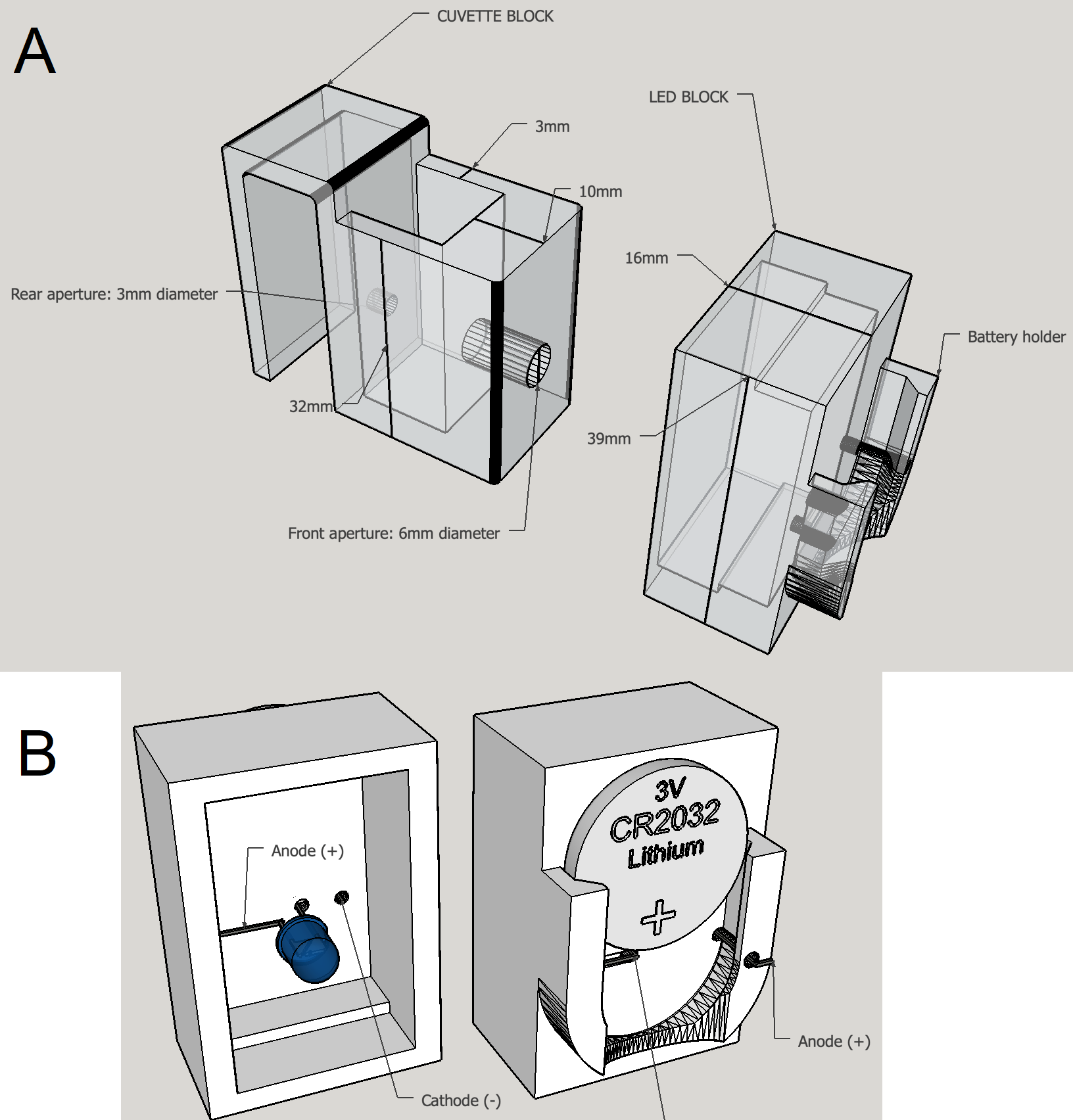


S1B Text. (A) X-Ray view of the two blocks of the Phone Spectrophotometer (B) LED mounted in the printed block, with a CR2032 battery. The anode passes through the right hole to the outside, touching the battery when it is in the down position and the cathode passes through the middle hole to the outside and them through the further left hole to the inside of the model, creating a loop

After the 3D modelling we proceeded to the slicing software Ultimaker Cura in order to generate a G-code file (file with instructions needed for the 3D printer), the slicer gets its name from its inherent characteristic: it divides the object with layers (slices) each one with a specified layer height. Many 3D printing characteristics may be changed within Ultimaker Cura, and the most relevant used in the 3D printing of our models are described in Table SI1.

Table SI1 - Main characteristics used to 3D print the Phone Spectrophotometer

| **Characteristic** | **Selection** |
| --- | --- |
| Layer height | 0.2mm |
| Wall thickness | 2mm |
| Top/Bottom thickness | 0.8mm |
| Infill density | 20% |
| Printing temperature | 210ºC |
| Retraction | Enabled |
| Print speed | 50mm/s |
| Generate support | No |
| Build plate adhesion type | Brim |

The 3D printer used was a RepRap Micro Delta, that use a fused deposition modelling process, and a Traffic Black PLA filament (RAL 9017), from Fillamentum Manufacturing Czech s.r.o.. Very little post-processing was needed after the printing process, mostly due to a little excess of the fused plastic material.

S1C Text shows the absorbance spectra of the molecules used to construct calibration graphs. The absorbance spectra were measured using Pasco 2600 spectrophotometer.


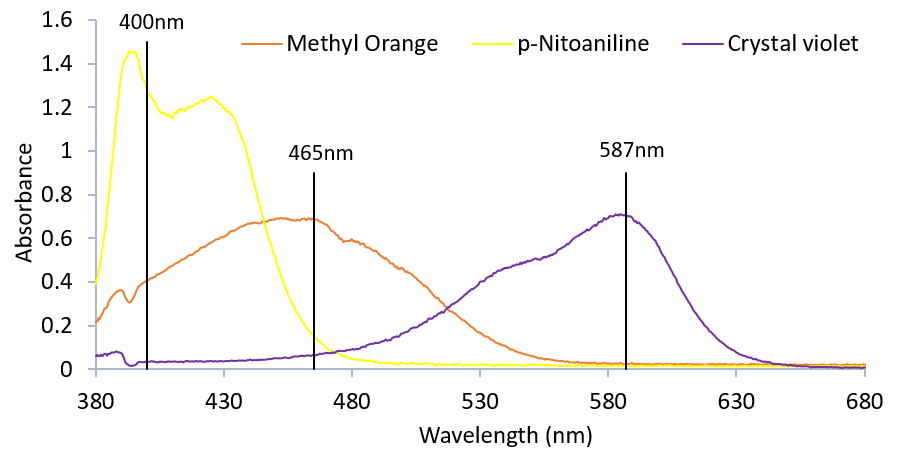


S1C Text. Absorbance spectra of Methyl Orange (orange line), Crystal Violet (Purple line) and p-Nitroaniline (yellow line). Data available in S1 Data

As shown in S1D Text, inclusion of a resistor (165Ω) made the intensity of light emitted over a prolonged period more stable. The intensity was lower when the resistor was included, but did not prevent the use in the smartphone spectrophotometer. Even with a resistor, if taking absorbance measurements over time, without recalibrating, it would be worth leaving the LED on for 20mins before use to reach the most stable emission intensity. There is space for a resistor between the two blocks of the presented model and a model with resistor shown on GitHub (<https://github.com/VascoRibeiroPereira/phone-spectrophotometer/blob/master/Issues/Adding%20a%20Resistor.md>).


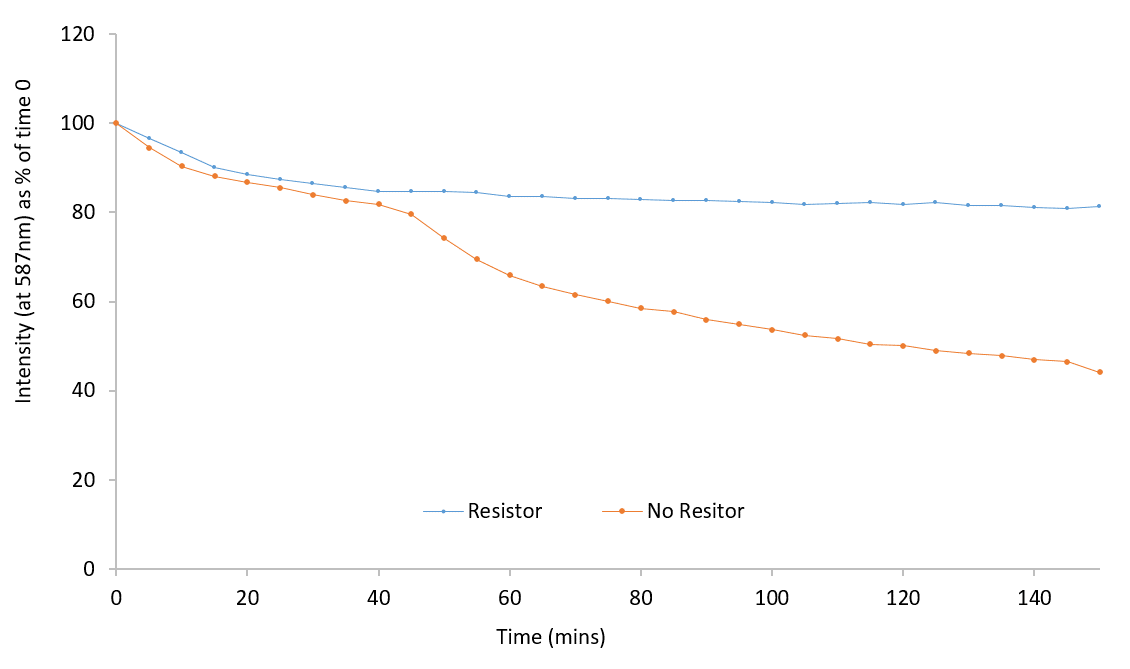


S1D Text. Effect of a resistor on intensity of light over time. Intensity is expressed as a % of the initial, 0 min measurement. Data available in S1 Data

.

**References**

1. Wego A. Accuracy simulation of an LED based spectrophotometer. Optik; 2013; 124: 644-649. doi:10.1016/j.ijleo.2012.01.005.

# 2. Jenway. 6300 and 6320D Visible and 6305 UV/Vis Spectrophotometers technical specification. 2019; <http://www.jenway.com/product.asp?dsl=289>

3. Jenway. 7310 and 7315 Visible Spectrophotometers technical specification. 2019; <http://www.jenway.com/product.asp?dsl=563>
